# X-ray screening identifies active site and allosteric inhibitors of SARS-CoV-2 main protease

**DOI:** 10.1126/science.abf7945

**Published:** 2021-04-02

**Authors:** Sebastian Günther, Patrick Y. A. Reinke, Yaiza Fernández-García, Julia Lieske, Thomas J. Lane, Helen M. Ginn, Faisal H. M. Koua, Christiane Ehrt, Wiebke Ewert, Dominik Oberthuer, Oleksandr Yefanov, Susanne Meier, Kristina Lorenzen, Boris Krichel, Janine-Denise Kopicki, Luca Gelisio, Wolfgang Brehm, Ilona Dunkel, Brandon Seychell, Henry Gieseler, Brenna Norton-Baker, Beatriz Escudero-Pérez, Martin Domaracky, Sofiane Saouane, Alexandra Tolstikova, Thomas A. White, Anna Hänle, Michael Groessler, Holger Fleckenstein, Fabian Trost, Marina Galchenkova, Yaroslav Gevorkov, Chufeng Li, Salah Awel, Ariana Peck, Miriam Barthelmess, Frank Schlünzen, P. Lourdu Xavier, Nadine Werner, Hina Andaleeb, Najeeb Ullah, Sven Falke, Vasundara Srinivasan, Bruno Alves França, Martin Schwinzer, Hévila Brognaro, Cromarte Rogers, Diogo Melo, Joanna J. Zaitseva-Doyle, Juraj Knoska, Gisel E. Peña-Murillo, Aida Rahmani Mashhour, Vincent Hennicke, Pontus Fischer, Johanna Hakanpää, Jan Meyer, Philip Gribbon, Bernhard Ellinger, Maria Kuzikov, Markus Wolf, Andrea R. Beccari, Gleb Bourenkov, David von Stetten, Guillaume Pompidor, Isabel Bento, Saravanan Panneerselvam, Ivars Karpics, Thomas R. Schneider, Maria Marta Garcia-Alai, Stephan Niebling, Christian Günther, Christina Schmidt, Robin Schubert, Huijong Han, Juliane Boger, Diana C. F. Monteiro, Linlin Zhang, Xinyuanyuan Sun, Jonathan Pletzer-Zelgert, Jan Wollenhaupt, Christian G. Feiler, Manfred S. Weiss, Eike-Christian Schulz, Pedram Mehrabi, Katarina Karničar, Aleksandra Usenik, Jure Loboda, Henning Tidow, Ashwin Chari, Rolf Hilgenfeld, Charlotte Uetrecht, Russell Cox, Andrea Zaliani, Tobias Beck, Matthias Rarey, Stephan Günther, Dusan Turk, Winfried Hinrichs, Henry N. Chapman, Arwen R. Pearson, Christian Betzel, Alke Meents

**Affiliations:** 1 Center for Free-Electron Laser Science, Deutsches Elektronen-Synchrotron DESY, Notkestr. 85, 22607 Hamburg, Germany.; 2 Bernhard Nocht Institute for Tropical Medicine, Bernhard-Nocht-Str. 74, 20359 Hamburg, Germany.; 3 Diamond Light Source Ltd., Diamond House, Harwell Science and Innovation Campus, Didcot, OX11 0DE, UK.; 4 Universität Hamburg, Center for Bioinformatics, Bundesstr. 43, 20146 Hamburg, Germany.; 5 Hamburg Centre for Ultrafast Imaging, Universität Hamburg, Luruper Chaussee 149, 22761 Hamburg, Germany.; 6 Universität Hamburg, Institut für Nanostruktur- und Festkörperphysik, Luruper Chaussee 149, 22761 Hamburg, Germany.; 7 European XFEL GmbH, Holzkoppel 4, 22869 Schenefeld, Germany.; 8 Heinrich Pette Institute, Leibniz Institute for Experimental Virology, Martinistr. 52, 20251 Hamburg, Germany.; 9 Max Planck Institute for Molecular Genetics, Ihnestr. 63-73, 14195 Berlin, Germany.; 10 Universität Hamburg, Department of Chemistry, Institute of Physical Chemistry, Grindelallee 117, 20146 Hamburg, Germany.; 11 Max Planck Institute for the Structure and Dynamics of Matter, Luruper Chaussee 149, 22761 Hamburg, Germany.; 12 Department of Chemistry, UC Irvine, Irvine, CA 92697-2025, USA.; 13 Deutsches Elektronen-Synchrotron DESY, Notkestr. 85, 22607, Hamburg, Germany.; 14 Vision Systems, Hamburg University of Technology, 21071 Hamburg, Germany.; 15 Division of Biology and Biological Engineering, California Institute of Technology, Pasadena, CA 91125, USA.; 16 Universität Hamburg, Department of Chemistry, Institute of Biochemistry and Molecular Biology and Laboratory for Structural Biology of Infection and Inflammation, c/o DESY, 22607 Hamburg, Germany.; 17 Fraunhofer Institute for Translational Medicine and Pharmacology and Fraunhofer Cluster of Excellence for Immune Mediated Diseases, Schnackenburgallee 114, 22525 Hamburg, Germany.; 18 Dompé Farmaceutici SpA, 67100 L’Aquila, Italy.; 19 EMBL Outstation Hamburg, c/o DESY, Notkestr. 85, 22607 Hamburg, Germany.; 20 Institute of Molecular Medicine, University of Lübeck, 23562 Lübeck, Germany.; 21 Hauptmann Woodward Medical Research Institute, 700 Ellicott Street, Buffalo, NY 14203, USA.; 22 German Center for Infection Research, Hamburg-Lübeck-Borstel-Riems Site, University of Lübeck, 23562 Lübeck, Germany.; 23 Helmholtz Zentrum Berlin, Macromolecular Crystallography, Albert-Einstein-Str. 15, 12489 Berlin, Germany.; 24 Department of Biochemistry and Molecular and Structural Biology, Jozef Stefan Institute, Jamova 39, 1000 Ljubljana, Slovenia.; 25 Centre of Excellence for Integrated Approaches in Chemistry and Biology of Proteins, Jamova 39, 1000 Ljubljana, Slovenia.; 26 Universität Hamburg, Department of Chemistry, Institute of Biochemistry and Molecular Biology, Martin-Luther-King-Platz 6, 20146 Hamburg, Germany.; 27 Research Group for Structural Biochemistry and Mechanisms, Department of Structural Dynamics, Max Planck Institute for Biophysical Chemistry, Am Fassberg 11, 37077 Göttingen, Germany.; 28 Institute for Organic Chemistry and BMWZ, Leibniz University of Hannover, Schneiderberg 38, 30167 Hannover, Germany.; 29 Universität Greifswald, Institute of Biochemistry, Felix-Hausdorff-Str. 4, 17489 Greifswald, Germany.; 30 Universität Hamburg, Department of Physics, Luruper Chaussee 149, 22761 Hamburg, Germany.

## Abstract

The coronavirus disease (COVID-19) caused by SARS-CoV-2 is creating tremendous human suffering. To date, no effective drug is available to directly treat the disease. In a search for a drug against COVID-19, we have performed a high-throughput x-ray crystallographic screen of two repurposing drug libraries against the SARS-CoV-2 main protease (M^pro^), which is essential for viral replication. In contrast to commonly applied x-ray fragment screening experiments with molecules of low complexity, our screen tested already-approved drugs and drugs in clinical trials. From the three-dimensional protein structures, we identified 37 compounds that bind to M^pro^. In subsequent cell-based viral reduction assays, one peptidomimetic and six nonpeptidic compounds showed antiviral activity at nontoxic concentrations. We identified two allosteric binding sites representing attractive targets for drug development against SARS-CoV-2.

## A large-scale screen to target SARS-CoV-2

The severe acute respiratory syndrome coronavirus 2 (SARS-CoV-2) genome is initially expressed as two large polyproteins. Its main protease, M^pro^, is essential to yield functional viral proteins, making it a key drug target. Günther *et al.* used x-ray crystallography to screen more than 5000 compounds that are either approved drugs or drugs in clinical trials. The screen identified 37 compounds that bind to M^pro^. High-resolution structures showed that most compounds bind at the active site but also revealed two allosteric sites where binding of a drug causes conformational changes that affect the active site. In cell-based assays, seven compounds had antiviral activity without toxicity. The most potent, calpeptin, binds covalently in the active site, whereas the second most potent, pelitinib, binds at an allosteric site.

*Science*, this issue p. 642

## Teaser

A repurposed drug-library screen reveals two allosteric drug binding sites of the SARS-CoV-2 main protease.

**Fig. 1:**
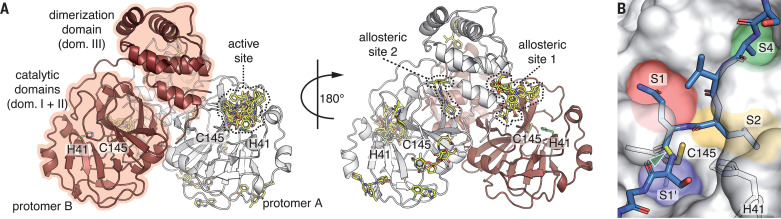
The x-ray screening of drug-repurposing libraries reveals compound binding sites distributed across the complete M^pro^ surface. (**A**) Schematic drawing of M^pro^ dimer structure. Protomer A is shown in white, and protomer B is in red. For clarity, the 29 binding compounds (yellow sticks) are only depicted on one of the two protomers. Catalytic residues His^41^ (H41) and Cys^145^(C145), the active site, and two allosteric drug binding sites are highlighted. (**B**) Close-up view of the active site with peptide substrate bound (blue sticks), modeled after SARS-CoV M^pro^ (PDB 2Q6G). The scissile bond is indicated in yellow and with the green arrowhead. Substrate binding pockets S1ʹ, S1, S2, and S4 are indicated by colored regions.

Infection of host cells by SARS-CoV-2 is governed by the complex interplay of molecular factors from both the host and the virus (*1*, *2*). Coronaviruses are RNA viruses with a genome of approximately 30,000 nucleotides. The viral open reading frames are expressed as two overlapping large polyproteins which must be separated into functional subunits for replication and transcription activity (*1*). This proteolytic cleavage is primarily accomplished by the main protease (M^pro^), also known as 3C-like protease 3CL^pro^ or nsp5. M^pro^ cleaves the viral polyprotein pp1ab at 11 distinct sites. The core cleavage motif is Leu-Gln↓(Ser/Ala/Gly) (*1*). M^pro^ possesses a chymotrypsin-like fold appended with a C-terminal helical domain and harbors a catalytic dyad comprised of Cys^145^ and His^41^ in its active site, which is formed by four major pockets that are labeled according to their position relative to the scissile bond of the substrate (Fig. 1) (*1*). The active site is located in a cleft between the two N-terminal domains of the three-domain structure of the monomer, whereas the C-terminal helical domain is involved in regulation and dimerization of the enzyme (Fig. 1A). Because of its central involvement in virus replication, M^pro^ is recognized as a prime target for antiviral drug discovery and compound screening activities aiming to identify and optimize drugs which can tackle coronavirus infections (*3*). Indeed, a number of recent publications confirm the potential of targeting M^pro^ for inhibition of virus replication (*1*, *2*, *4*).

In order to find drug candidates against SARS-CoV-2, we performed a large-scale x-ray crystallographic screen of M^pro^ against two repurposing libraries containing 5953 compounds from the Fraunhofer IME Repurposing Collection and the Safe-in-man library from Dompé Farmaceutici S.p.A. (*5*).

In contrast to crystallographic fragment screening experiments, compounds in repurposing libraries are chemically more complex (fig. S1A) (*6*, *7*). Thus, these compounds likely bind more specifically and with higher affinity (*8*). Because of the higher molecular weights, we performed cocrystallization experiments at a physiological pH of 7.5 instead of compound soaking into native crystals (*9*).

From the 5953 compounds in our screen, we obtained x-ray diffraction datasets for 2381 compounds, which we subjected to automated structure refinement followed by cluster analysis (*10*) and pan dataset density analysis (PanDDA) (*11*) (table S1). We observed additional electron density, indicating binding to M^pro^, for 43 compounds, which were classified as hits, representing 37 distinct compounds (tables S1, S2, and S3). From these, the binding mode could be unambiguously determined for 29 molecules (Fig. 1A and table S4). The majority of hits were found in the active site of the enzyme. Of the 16 active site binders, six covalently bind as thioethers to Cys^145^, one compound binds covalently as a thiohemiacetal to Cys^145^, one is zinc-coordinated, and eight bind noncovalently. The remaining 13 compounds bind outside the active site at various locations (Fig. 1A).

**Fig. 2:**
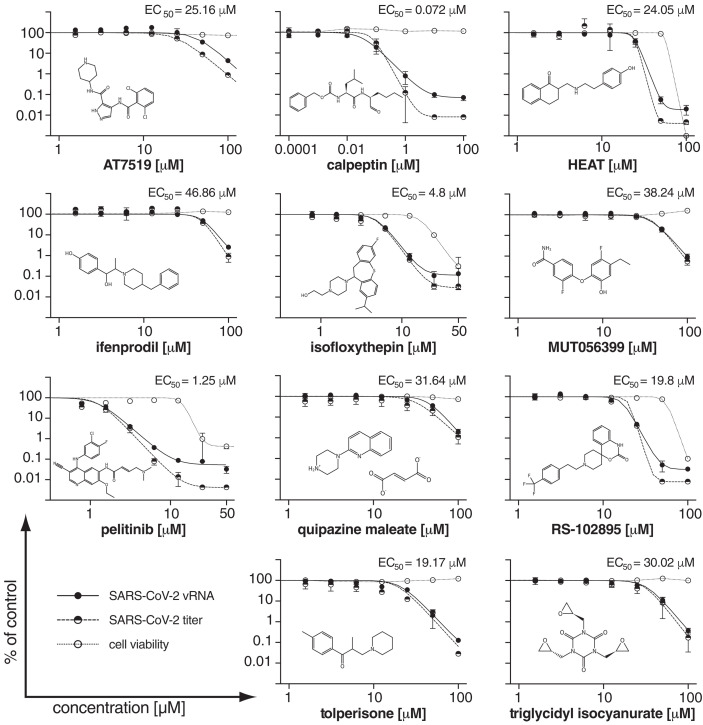
Effect of selected compounds on SARS-CoV-2 replication in Vero E6 cells. The vRNA yield (solid circles), viral titers (half-solid circles), and cell viability (empty circles) were determined by reverse transcription–quantitative polymerase chain reaction, immunofocus assays, and the CCK-8 method, respectively. EC_50_ for the viral titer reduction is shown. Individual data points represent means ± SD from three independent replicates in one experiment.

Of the 43 hits from our x-ray screen, 37 compounds were available in quantities required for testing their antiviral activity against SARS-CoV-2 in cell assays (table S2). Nine compounds that reduced viral RNA (vRNA) replication by at least two orders of magnitude in Vero E6 cells (fig. S2) were further evaluated to determine the effective concentrations that reduced not only vRNA but also SARS-CoV-2 infectious particles by 50% (EC_50_) (Fig. 2). Additionally, AT7519 and ifenprodil, which showed slightly lower vRNA level reduction, were included because of their distinct binding sites outside of the active site. From these 11, seven compounds (AT7519, calpeptin, ifenprodil, MUT056399, pelitinib, tolperisone, and triglycidyl isocyanurate) exhibited a ≥100-fold reduction in infectious particles in combination with either a selectivity index [SI; calculated as the 50% cytotoxic concentration (CC_50_) divided by the EC_50_] of >5 or no cytotoxicity in the tested concentration range and are considered antivirally active (table S5).

Here, we focus on a more detailed description of the 11 compounds analyzed in the secondary screen, which are grouped according to their different binding sites. The remaining hits are described in the supplementary text and figs. S3 to S5.

**Fig. 3:**
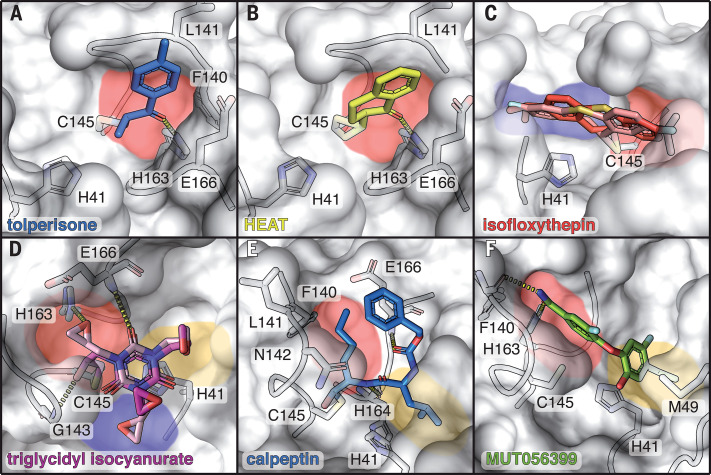
Covalent and noncovalent binders in the active site of M^pro^. Bound compounds are depicted as colored sticks, and the surface of M^pro^ is shown in gray with selected interacting residues shown as sticks. Substrate binding pockets are colored as in Fig. 1. Hydrogen bonds are depicted by dashed lines. (**A**) Tolperisone. (**B**) HEAT. (**C**) Isofloxythepin. (**D**) Triglycidyl isocyanurate. (**E**) Calpeptin. (**F**) MUT056399.

Tolperisone, 2-[β-(4-hydroxyphenyl)-ethylaminomethyl]-tetralone (HEAT), and isofloxythepin bind covalently to the active site. Tolperisone is antivirally active (EC_50_ = 19.17 μM) and shows no cytotoxicity (CC_50_ > 100 μM) (Fig. 2), whereas HEAT (EC_50_ = 24.05 μM, CC_50_ = 55.42 μM) and isofloxythepin (EC_50_ = 4.8 μM, CC_50_ = 17 μM) show unfavorable cytotoxicity. For all three compounds, only breakdown products are observed in the active site. Tolperisone and HEAT are β-aminoketones, but we only observe the part of the drug containing the ketone (2,4′-dimethylpropiophenone and 2-methyl-1-tetralone), whereas the remaining part with the amine group is missing. The breakdown product binds as a Michael acceptor to the thiol of Cys^145^, independently confirmed for HEAT by mass spectrometry (fig. S6 and table S6). The decomposition of tolperisone and HEAT was detected in both the crystallization and cell culture conditions (fig. S7) and is reported to be pH dependent (*12*). The parent compounds can be regarded as prodrugs (*13*, *14*). In the x-ray structures the aromatic ring systems of tolperisone (Fig. 3A) and HEAT (Fig. 3B) protrude into the S1 pocket and form van der Waals contacts with the backbone of Phe^140^ and Leu^141^ and the side chain of Glu^166^. In addition, the keto group accepts a hydrogen bond from the imidazole side chain of His^163^. Tolperisone is used as a skeletal muscle relaxant (*15*). The x-ray structure suggests that isofloxythepin binds similarly as a fragment to Cys^145^ (Fig. 3C).

Triglycidyl isocyanurate has antiviral activity (EC_50_ = 30.02 μM, CC_50_ > 100 μM) and adopts covalent and noncovalent binding modes to the active site. In both modes, the compound’s central ring sits on top of the catalytic dyad (His^41^, Cys^145^), and its three epoxypropyl substituents reach into subsites S1′, S1, and S2. The noncovalent binding mode is stabilized by hydrogen bonds to the main chain of Gly^143^ and Gln^166^ and to the side chain of His^163^. In the covalently bound form, one oxirane ring is opened by nucleophilic attack of Cys^145^, forming a thioether (Fig. 3D). Triglycidyl isocyanurate has been tested as an antitumor agent (*16*).

Calpeptin shows the highest antiviral activity in the screen (EC_50_ = 72 nM, CC_50_ > 100 μM). It binds covalently via its aldehyde group to Cys^145^, forming a thiohemiacetal. This peptidomimetic inhibitor occupies substrate pockets S1 to S3, similar to the peptidomimetic inhibitors GC-376 (*17*, *18*), calpain inhibitors (*19*), N3 (*2*), and the α-ketoamide 13b (*1*). The peptidomimetic backbone forms hydrogen bonds to the main chain of His^164^ and Glu^166^, whereas the norleucine side chain maintains van der Waals contacts with the backbone of Phe^140^, Leu^141^, and Asn^142^ (Fig. 3E). Calpeptin has known activity against SARS-CoV-2 M^pro^ in enzymatic assays (*17*). The structure is highly similar to the common protease inhibitor leupeptin (fig. S3A), which served as a positive control in our x-ray screen but was not tested further in antiviral assays*.* In silico docking experiments also suggested calpeptin as a possible M^pro^ binding molecule (table S7). Calpeptin also inhibits cathepsin L (*20*), and dual targeting of cathepsin L and M^pro^ is suggested as an attractive path for SARS-CoV-2 inhibition (*19*).

MUT056399 binds noncovalently to the active site (EC_50_ = 38.24 μM, CC_50_ > 100 μM). The diphenyl ether core of MUT056399 blocks access to the catalytic site, which consists of Cys^145^ and His^41^. The terminal carboxamide group occupies pocket S1 and forms hydrogen bonds to the side chain of His^163^ and the backbone of Phe^140^ (Fig. 3F). The ethyl phenyl group of the molecule reaches deep into pocket S2, which is enlarged by a shift of the side chain of Met^49^ out of the substrate binding pocket. MUT056399 was developed as an antibacterial agent against multidrug-resistant *Staphylococcus aureus* strains (*21*).

Quipazine maleate showed moderate antiviral activity (EC_50_ = 31.64 μM, CC_50_ > 100 μM). In the x-ray structure, only the maleate counterion is observed covalently bound as a thioether (supplementary text and fig. S3B). Maleate is observed in structures of six other compounds showing no antiviral activity. The observed antiviral activity is thus likely caused by an off-target effect of quipazine.

In general, the enzymatic activity of M^pro^ relies on the architecture of the active site, which critically depends on the dimerization of the enzyme and the correct relative orientation of the subdomains. This could allow ligands that bind outside of the active site to affect activity. In fact, we identified two such allosteric binding sites of M^pro^_._

**Fig. 4:**
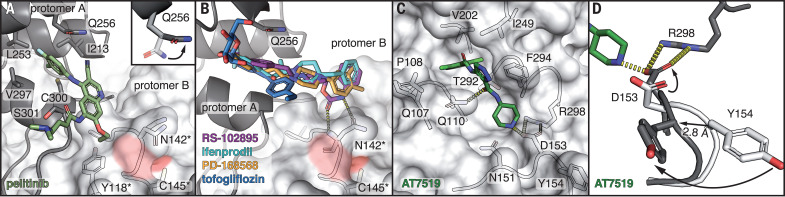
Screening hits at allosteric sites of M^pro^. (**A**) Close-up view of the binding site in the dimerization domain (protomer A, gray cartoon representation), close to the active site of the second protomer (protomer B, surface representation) in the native dimer. Residues forming the hydrophobic pocket are indicated. Pelitinib (dark green) binds to the C-terminal α-helix at Ser^301^ and pushes against Asn^142^ and the β-turn of the pocket S1 of protomer B (residues marked with an asterisk). The inset shows the conformational change of Gln^256^ (gray sticks) compared with the M^pro^ apo structure (white sticks). (**B**) RS-102895 (purple), ifenprodil (cyan), PD-168568 (orange), and tofogliflozin (blue) occupy the same binding pocket as pelitinib. (**C**) AT7519 occupies a deep cleft between the catalytic and dimerization domain of M^pro^. (**D**) Conformational changes in the AT7519-bound M^pro^ structure (gray) compared with those in the apo structure (white).

Five compounds of our x-ray screen bind in a hydrophobic pocket in the C-terminal dimerization domain (Fig. 4, A and B), located close to the oxyanion hole in pocket S1 of the substrate binding site. One of these showed strong antiviral activity (Fig. 2). Another compound binds between the catalytic and dimerization domains of M^pro^.

Central to the first allosteric binding site is a hydrophobic pocket formed by Ile^213^, Leu^253^, Gln^256^, Val^297^, and Cys^300^ within the C-terminal dimerization domain (Fig. 4A). Pelitinib, ifenprodil, RS-102895, PD-168568, and tofogliflozin all exploit this site by inserting an aromatic moiety into this pocket.

Pelitinib shows the second highest antiviral activity in our screen (EC_50_ = 1.25 μM, CC_50_ = 13.96 μM). Its halogenated benzene ring binds to the hydrophobic groove in the helical domain, which becomes accessible by movement of the Gln^256^ side chain (Fig. 4A). The central 3-cyanoquinoline moiety interacts with the end of the C-terminal helix (Ser^301^). The ethyl ether substituent pushes against Tyr^118^ and Asn^142^ (from loop 141–144 of the S1 pocket) of the opposing protomer within the native dimer. The integrity of this pocket is crucial for enzyme activity (*22*). Pelitinib is an amine-catalyzed Michael acceptor (*23*) and was developed as an anticancer agent to bind to a cysteine in the active site of the tyrosine kinase epidermal growth factor receptor inhibitor (*24*). However, from its observed binding position, it is impossible for it to reach into the active site, and no evidence for covalent binding to Cys^145^ is found in the electron density maps.

Ifenprodil and RS-102895 bind to the same hydrophobic pocket in the dimerization domain as pelitinib (Fig. 4B; fig. S4, A and B; and supplementary text). Only ifenprodil (EC_50_ = 46.86 μM, CC_50_ > 100 μM) shows moderate activity. RS-102895 (EC_50_ = 19.8 μM, CC_50_ = 54.98 μM) interacts, similar to pelitinib, with the second protomer by forming two hydrogen bonds to the side and main chains of Asn^142^, whereas the other compounds exhibit weaker or no interaction with the second protomer. PD-168568 and tofogliflozin bind the same site but are inactive (Fig. 4B and fig. S4, C and D).

The second allosteric site is formed by the deep groove between the catalytic domains and the dimerization domain. AT7519 is the only compound in our screen that we identified bound to this site (Fig. 4C). Though it has only moderate activity, we discuss it here because this site may be a target. The chlorinated benzene ring is engaged in various van der Waals interactions to loop 107-110, Val^202^, and Thr^292^. The central pyrazole has van der Waals contacts to Ile^249^ and Phe^294^, and its adjacent carbonyl group forms a hydrogen bond to the side chain of Gln^110^. The terminal piperidine sits on top of Asn^151^ and forms hydrogen bonds to the carboxylate of Asp^153^. This results in a displacement of loop 153-155, slightly narrowing the binding groove. The Cα atom of Tyr^154^ moves 2.8 Å, accompanied by a conformational change of Asp^153^ (Fig. 4D). This allows hydrogen bonding to the compound and the formation of a salt bridge to Arg^298^. Arg^298^ is crucial for dimerization (*25*). The mutation Arg^298^Ala causes a reorientation of the dimerization domain relative to the catalytic domain, leading to changes in the oxyanion hole and destabilization of the S1 pocket by the N terminus. AT7519 was evaluated for treatment of human cancers (*26*). The potential of allosteric inhibition of M^pro^ through modulation of Arg^298^ has been independently demonstrated by mass spectrometry (*27*).

Our x-ray screen revealed 43 compounds binding to M^pro^, with seven compounds showing antiviral activity against SARS-CoV-2. We present structural evidence for interaction of these compounds at active and allosteric sites of M^pro^, although we cannot exclude that off-target effects played a role in the antiviral effect in cell culture, in particular for compounds with a low selectivity index. Conversely, an absence of antiviral activity of compounds binding clearly to M^pro^ in the crystal might be due to rapid metabolization in the cellular environment. Calpeptin and pelitinib showed strong antiviral activity with low cytotoxicity and are suitable for preclinical evaluation. In any case, all hit compounds are valuable lead structures with potential for further drug development, especially because drug-repurposing libraries offer the advantage of proven bioactivity and cell permeability (*28*).

The most active compound, calpeptin, binds in the active site similar to other members of the large class of peptide-based inhibitors that bind as thiohemi-acetals or -ketals to M^pro^ (*29*). In addition to this peptidomimetic inhibitor, we discovered several nonpeptidic inhibitors. Those compounds binding to the active site of M^pro^ contained new Michael acceptors based on β-aminoketones (tolperisone and HEAT). These compounds lead to the formation of thioethers and have not been described as prodrugs for viral proteases. We also identified a noncovalent binder, MUT056399, that blocked the active site. In addition to this common active site inhibition, we identified compounds that inhibit the enzyme through binding at two allosteric sites of M^pro^.

The first allosteric site (dimerization domain) is in the direct vicinity of the S1 pocket of the adjacent monomer within the native dimer. The potential for antiviral inhibition through this site is demonstrated by pelitinib. The hydrophobic nature of the residues forming the main pocket is conserved in all human coronavirus M^pro^ (fig. S8). Consequently, potential drugs targeting this binding site may be effective against other coronaviruses. The potential of the second allosteric site as a druggable target is demonstrated by the observed moderate antiviral activity of AT7519.

## Supplementary Materials

science.sciencemag.org/content/372/6542/642/suppl/DC1 Materials and Methods Supplementary Text Figs. S1 to S9 Tables S1 to S7 References (*30*–*54*) MDAR Reproducibility Checklist
